# Persistent ciprofloxacin exposure induced the transformation of Klebsiella pneumoniae small colony variant into mucous phenotype

**DOI:** 10.3389/fcimb.2023.1259296

**Published:** 2023-10-19

**Authors:** Hua Zou, Qian Li, Yan Su, Lei Zhang, Xinyuan Zhang, Chunli Li

**Affiliations:** Department of Laboratory Medicine, Chongqing Health Center for Women and Children, Women and Children’s Hospital of Chongqing Medical University, Chongqing, China

**Keywords:** small colony variant, Klebsiella pneumoniae, subinhibitory concentrations of antibiotics, Ciprofloxacin, *umuC*, IcmK

## Abstract

**Introduction:**

Small colony variant (SCV) is a bacterial phenotype closely related to persistent and recurrent infections. SCVs are mutations that occur within bacterial populations, resulting in a change in bacterial morphology and the formation of small colonies. This morphological change may enhance bacterial resistance to antibiotics and contribute to persistent and recurrent infections.

**Methods:**

We isolated Klebsiella pneumoniae (KPN) and its SCV from a child with recurrent respiratory tract infections. KPN and SCV were treated with subinhibitory concentrations of antibiotics. growth curves, serum resistance experiments, macrophage phagocytosis experiments and whole genome sequencing were used to characterize KPN and SCV.

**Results:**

After treating KPN and SCV with subinhibitory concentrations of antibiotics, we found that ciprofloxacin induced the SCV transition to the mucoid phenotype. We found that the growth of mucoid Klebsiella pneumoniae was significantly slower than maternal strain and SCV though growth curves. Serum resistance experiments showed that mucoid strains had significantly higher serum resistance compared to maternal strain and SCV. Macrophage phagocytosis experiments revealed that SCV had significantly higher intracellular survival rates compared to maternal strain and mucoid strains. Differential gene analysis of three strains revealed that the mucoid strain contained DNA polymerase V subunit UmuC gene on the plasmid, while the SCV strain had an additional IcmK family IV secretion protein on its plasmid.

**Discussion:**

Our study showed the SCV of KPN changed to a mucoid colony when exposed to subinhibitory concentrations of ciprofloxacin. The higher resistance of serum of mucoid colonies was possibly related to the UmuC gene, while the increased intracellular survival of SCV may be related to the IcmK family type IV secretion proteins.

## Introduction

Klebsiella pneumoniae (KPN*)* is a common cause of antimicrobial-resistant opportunistic infections in patients of all ages. KPN could cause a wide range of diseases including pneumonia, urinary tract infections (UTIs), bloodstream infections and sepsis (Effah et al., 2020). These infections are particularly a problem among neonates, elderly and immunocompromised individuals (Bengoechea and Sa Pessoa, 2019). The global emergence and spread of genes of antimicrobial resistance such as ESBL and carbapenemase genes in KPN isolates present a significant danger to public health. High mortality rates, extended hospitalization, coupled with high cost are often associated with infections caused by this organism (Wyres et al., 2020). It was previously thought that KPN generally caused acute infection, but recent studies have found that KPN can exist in the human body for a long time, causing persistent infection or recurrent infection (Zhong et al., 2018; Vachvanichsanong et al., 2020; Collingwood et al., 2023). In this study, KPN was isolated from a child with recurrent pneumonia, and we hypothesized that it was due to the long-term presence of KPN in the child’s respiratory tract leading to recurrent pulmonary infection. However, little research has been done on how KPN persists in the respiratory tract.

Small colony variants (SCVs) are a subgroup of bacteria with special phenotypic and pathogenic characteristics. The most typical feature of SCVs is slow growth, small colony size, and only about 1/10 the size of wild-type strains (Proctor et al., 2006). The first discoverers of SCV were Proctor, who reported the discovery of SCV in Staphylococcus aureus (S. aureus) in 1998 (Proctor et al., 1998). Since then, research on SCVs has been conducted. Further studies have found that many bacteria, such as S. aureus (Von Eiff, 2008), coagulase-negative staphylococci (Fernandez-Rodriguez et al., 2021), Pseudomonas aeruginosa (Anand et al., 2018; Besse et al., 2023), have the ability to form SCVs, and SCVs have been isolated from abscesses, blood, respiratory tract, and soft tissues.

In the process of SCV formation, long-term use of antibiotics and host immune mechanisms play a crucial role. Aminoglycosides, sulfonamides such as compound sulfamethoxazole, quinolones, and other antibiotic can induce the formation of SCVs (Proctor et al., 2006). The intracellular environment of eukaryotic cells also favors the selection and survival of SCVs (Tuchscherr et al., 2011). Increasingly, studies have shown a close association between SCVs and many chronic and recurrent infectious diseases especially SCV of S. aureus (Tuchscherr et al., 2011). Besier et al. showed that the emergence of S. aureus SCVs may be detrimental to the disease process and is related to the deterioration of lung disease in cystic fibrosis patients (Wolter et al., 2013). Compared with the parental strain, in the process of chronic infection, some important exotoxins such as hemolysin and plasma coagulase are significantly down-regulated in S. aureus SCVs, while the synthesis of components related to cell adhesion and biofilm formation is increased (Tuchscherr et al., 2011; Tuchscherr et al., 2015; Guo et al., 2022). Overall, the changes in virulence factor expression in SCVs can affect their ability to cause disease and evade the host immune response. Recent studies have shown that Pseudomonas aeruginosa can also form small colony variants and is closely associated with persistent infections in cystic fibrosis (Besse et al., 2023). Melissa et al. confirmed the causative role of a single *ispA* mutation in P. aeruginosa SCV emergence and increased aminoglycoside resistance. IspA is involved in the synthesis of ubiquinone, providing a possible link between electron transport and SCV formation in P. aeruginosa (Pitton et al., 2022).

SCV can be classified into two main types: stable and unstable SCVs. Stable SCVs have a stable genetic mutation that results in their small colony size and reduced metabolic activity, while unstable SCVs can revert to the wild-type phenotype under certain conditions. Clinical isolated SCVs often exhibit an unstable phenotype and can easily revert to the parental strain phenotype after passage (Guerillot et al., 2019). In this study, KPN was isolated from a child with recurrent lung infections, and after 48 hours of cultivation, pinpoint-sized colonies were found on blood agar plates. These colonies were identified as KPN by mass spectrometry. In order to maintain the phenotype of clinically isolated SCVs, we added sub-inhibitory concentrations of antibiotics and found that sub-inhibitory concentrations of ciprofloxacin induced the transition of SCV to mucoid-variant of KPN. However, to date, information regarding this phenomenon remains elusive. Better understanding of these data may enable effective treatment of patients at high risk.

## Method

### Bacterial strains

This study was performed in Chongqing Health Center for Women and Children in Southwest China. Isolate KPN from a sputum specimen of a child with recurrent pulmonary infections. After culturing on blood agar plates for 48 hours, a pinpoint-sized colony was picked.

### Antibiotics and *in vitro* susceptibility testing

All the isolates were identified at the species level by the VITEK MS (bioMerieux, Hazelwood, MO, United States) automated system, and routine antimicrobial susceptibility testing was performed by using the BD Phoenix™ system. At the same time, *K. pneumoniae* ATCC 700603 was selected as the standard strains for MIC detection. All standard strains were purchased from American Type Culture Collection and all antibiotics were purchased from Meilunbio in China.

### Subinhibitory concentration antibiotic experiment

The purpose of a subinhibitory concentration antibiotic experiment is to investigate the effects of antibiotics at concentrations that are lower than the minimum inhibitory concentration (MIC). Ceftazidime (CAZ), ceftriaxone (CRO), cefepime (FEP), ertapenem (ETP), imipenem (IPM), meropenem (MEM), piperacillin tazobactam (TZP), tigecycline (TGC), colistin (COL), levofloxacin (LEV), ciprofloxacin (CIP), amikacin (AMK) and gentamicin (GEN) were included. The selection of subinhibitory concentration dosage is based on two considerations: (1) not exceeding the MIC of the selected clinical isolate; (2) based on drug instructions or literature, it should be a concentration that can be actually achieved at the site of infection.

### Multilocus sequence typing and pulsed-field gel electrophoresis

Multilocus sequence typing (MLST) was performed by the amplifications of the internal fragments of seven housekeeping genes of parent strain, SCVs, and mucoid types of KPN isolates (*rpaB*, *gapA*, *mdh*, *pgi*, *phoE*, *infB* and *tonB*) according to the database (https://pubmlst.org/organisms). The clonal relationships of the isolates were further determined by pulsed-field gel electrophoresis (PFGE). Briefly, genomic DNAs of the CR-KP isolates were prepared in agarose blocks and digested with restriction enzyme *XbaI*. DNA fragments were separated using a CHEF II D-Mapper XA PFGE system with running conditions as described previously. The dendrogram of the PFGE profiles was clustered by the Dice coefficient and the unweighted pair group method (UPGAMA) on the basis of the dice similarity by the Quantity One software package 4.6 (Tenover et al., 1995). DNA patterns sharing ≥95% similarity (SA) was defined as Clusters (Contreras-Alvarado et al., 2021).

### Growth curve determination

The parent strain, SCVs, and mucoid type of KPN were streaked onto blood agar plates and incubated at 35°C for 24 hours. Several pure colonies were picked and inoculated into TSB and shaken overnight at 37°C, 150rpm. On the next day, the overnight culture was diluted 1:100 in fresh TSB. The diluted bacterial culture was measured for its absorbance value at a wavelength of 600nm using a UV spectrophotometer, which was recorded as the initial OD value (OD600). Subsequently, samples were taken at regular intervals to determine the OD value, and finally, the growth curve was plotted.

### Biofilm assay

Biofilms-quantification was performed as previously described. The strain was placed in LB liquid medium and shaken overnight at 37°C. From this culture, 10 μL of a bacterial suspension contained 1 × 10^6^ CFU/ml was used to inoculate 96-well polystyrene plates containing 90 μL of LB liquid, 37°C for 24 h. Subsequently, the medium was removed from the plates and each well was washed three times with phosphate buffer. After dyeing with 1% crystal violet for 15min, the dyeing solution was sucked out and cleaned with distilled water until the sucked liquid was colorless and dried. Finally, Crystal violet was fully dissolved with 20ul anhydrous ethanol and the absorbance value was measured at 570nm. The yield of biofilm formation of the strains was interpreted as follows: OD > 0.6 as strong-producing, 0.4 < OD ≤0.6 as moderate-producing and OD < 0.4 as weak-producing (Yan and Bassler, 2019).

### Serum resistance assay

The serum resistance assay is a laboratory technique used to evaluate the ability of bacteria to resist the bactericidal effects of serum. The parent strain, SCVs, and mucoid types of KPN were inoculated into TSB. Serum is collected from a healthy human source. The bacterial suspension is mixed with the serum and incubated for 4h. After incubation, the number of viable bacterial cells is determined by plating serial dilutions of the bacterial suspension onto agar plates and counting the number of colonies that grow (Uber et al., 2021).

### Macrophage survival assay

A macrophage survival assay is a laboratory technique used to study the ability of microorganisms to survive and replicate within macrophages. Centrifuge to collect logarithmic phase growth of parent strain, SCVs, and mucoid types of KPN and adjust it to 0.5 McFarland standard (1.5×10^8^ CFU/ml) with pre-cooled PBS. RAW264.7 cells were seeded in a 6-well cell culture plate and incubated until 70%-80% confluence. Then, parent strain, SCVs, and mucoid types of KPN were added to the cells at a MOI of 100:1 and incubated at 37°C and 5% CO^2^ for 1 hour. After washing with PBS for 3 times, fresh DMEM medium containing 10% fetal bovine serum was added, followed by treatment with 100 μg/ml gentamicin for 1 hour to kill extracellular bacteria. Each well was washed with 2 ml PBS for 3 times, and then, fresh DMEM medium containing 10% fetal bovine serum was added for another 4 hours of incubation. The infected cells were washed with PBS for 3 times to remove extracellular bacteria and dead cells. After that, the cells were treated with 0.5% Triton X-100 in PBS for 10 minutes, and the supernatant was diluted and plated onto TSB agar plates for overnight culture at 37°C. The differences in the colony counts of KPN in each group were observed (Campioni et al., 2021).

### Whole genome sequencing

The maternal strain, SCVs, and mucoid type of KPN were streaked on LB solid medium and cultured at 37°C for 12 h. Next, a single colony on the solid medium was inoculated into 200 mL of LB liquid medium and cultured at 37°C for approximately 12 h at 150 rpm. The cell biomass was harvested after 10 min centrifugation at 12,000 × g. Genomic DNA of maternal strain, SCVs, and mucoid type of KPN were extracted using Wizard® Genomic DNA Purification Kit (Promega) according to manufacture’s protocol. Purified genomic DNA was quantified by TBS-380 fluorometer (Turner BioSystems Inc., Sunnyvale, CA). High quality DNA (OD260/280 = 1.8~2.0, ≥10μg) was used to do further research. Genomic DNA was sequenced using a combination of PacBio Sequel II and Illumina sequencing platforms. The Illumina data was used to evaluate the complexity of the genome. The data generated from PacBio and Illumina platform were used for bioinformatics analysis. All of the analyses were performed using the free online platform of Majorbio Cloud Platform (http://cloud.majorbio.com) from Shanghai Majorbio Bio-pharm Technology Co.,Ltd (Hyatt et al., 2010; Wick et al., 2017; Chan and Lowe, 2019).

### Ethical considerations

The data and samples analyzed in the present study were obtained in accordance with the standards and approved by Chongqing Health Center for Women and Children the Institutional Review Board and Biomedical Ethics Committee. For this study, samples were collected at the microbiology laboratory of our hospital, with no contact with the patient. This study was retrospective and there was no patient identification performed during data collection. Therefore, the ethics committee determined that informed consent was not required.

## Results

### General Characteristics and antimicrobial susceptibility of maternal strain, SCVs, and mucoid type of KPN

**Figure 1:**
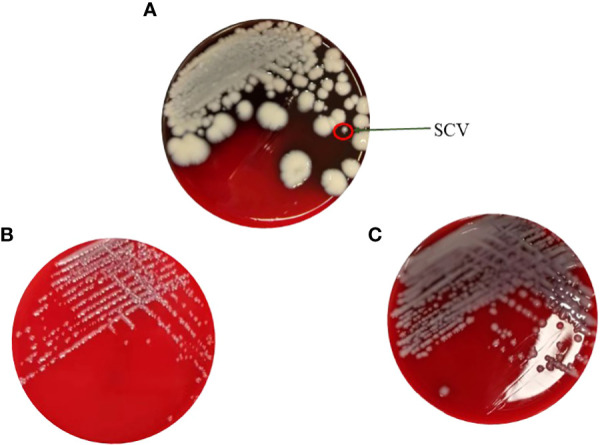
**(A)** The maternal strain and SCVs on the blood plate. **(B)** Pure cultivation of SCV with sub inhibitory concentrations of antibiotics. **(C)** Mucus type in the medium containing Ciprofloxacin with sub inhibitory concentration after 30 passages.

As shown in **Figure 1A**, KPN was isolated from a child with recurrent lung infections in this study. After placing the plate for 48 hours, needle like colonies appeared on the blood plate, which was identified as KPN by mass spectrometry. Single SCV was selected for pure cultivation for 24 hours, and its colony morphology was restored to the size of the mother colony. Placing SCV in a culture medium with sub inhibitory concentrations of antibiotics can maintain its morphology as shown in **Figure 1B**. After 30 passages, the small colony mutant transformed into mucus type in the medium containing Ciprofloxacin with sub inhibitory concentration (**Figure 1C**).

**Figure 2:**
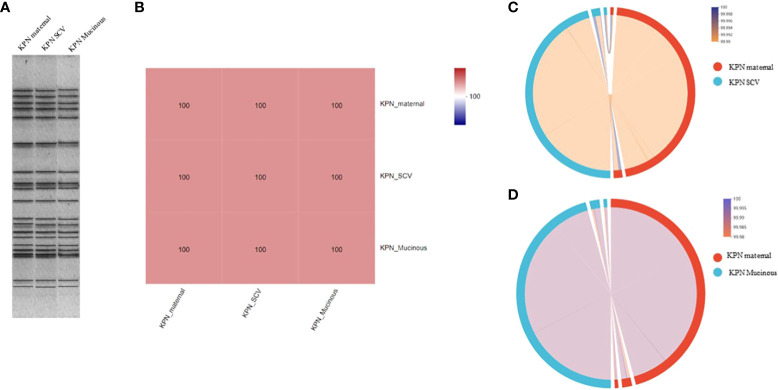
**(A)** PFGE dendrogram of maternal strain, SCVs and mucoid type of KPN. All the strains belong to ST792. **(B)** Average Aminoacid Identity of maternal strain, SCVs and mucoid type of KPN. **(C)** Collinearity analysis of maternal strain and SCVs. **(D)** Collinearity analysis of maternal strain and mucoid type of KPN.

For the antimicrobial susceptibility of these isolates, our study showed that maternal strain, SCVs and mucoid type of KPN were resistant to Cephalosporin and sensitive to Carbapenem, β-lactam/β-lactamase inhibitors, Quinolone and Aminoglycoside (**Table 1**). MLST results show that maternal strain, SCVs and mucoid type of KPN belong to ST792. PFGE, Average Aminoacid Identity analysis and Collinearity analysis showed that the three strains had high homology (**Figure 2**).

**Table 1:** Antimicrobial Susceptibility of CR-KP Isolates maternal strain, SCVs, and mucoid type of KPN.

Strains	Minimum inhibitory concentration (μg/mL)													
	IMP	ETP	MEM	FEP	CRO	CAZ	ATM	LEV	CIP	Amk	Gen	TZP	TEC	COL
maternal strain	0.25	0.5	0.06	64	64	16	32	0.5	0.25	0.5	2	0.5/4	0.5	<=0.5
SCVs	0.25	0.5	0.06	64	64	16	32	0.5	0.25	0.5	2	0.5/4	0.5	<=0.5
mucoid type	0.25	0.5	0.06	64	64	16	32	0.5	0.25	0.5	2	0.5/4	0.5	<=0.5

CAZ, ceftazidime; CRO, ceftriaxone; FEP, cefepime; ETP, ertapenem; IPM, imipenem; MEM, meropenem; TZP, piperacillin tazobactam; TC, tigecycline; FOS, colistin, fosfomycin; LEV, levofloxacin; CIP, ciprofloxacin; AMK, amikacin; GEN, gentamicin; TEC, Tigecycline; COL, colistin.

### Growth curve, biofilm formation, serum bactericidal assay and intracellular survival experiment of macrophages

**Figure 3:**
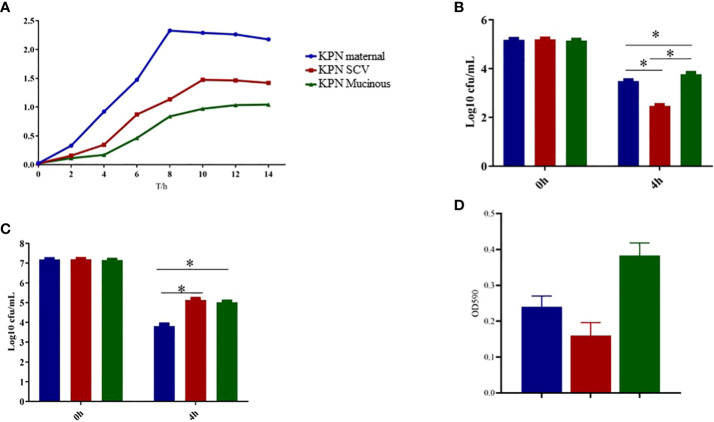
**(A)** Growth curve of maternal strain, SCVs and mucoid type of KPN. **(B)** Biofilm formation of maternal strain, SCVs and mucoid type of KPN **(C)** Serum bactericidal assay of maternal strain, SCVs and mucoid type of KPN **(D)** Intracellular survival experiment of macrophages of maternal strain, SCVs and mucoid type of KPN. *p<0.05.

As shown in **Figure 3A**, we measured the growth of the parental strain, SCV, and mucoid type in LB broth. The results showed that the growth rate of SCV and mucoid type colonies was significantly slower than that of the parental strain. Serum resistance assay results showed that mucoid type colonies had the strongest resistance to serum, followed by SCV, while the parental strain had the weakest resistance and was more susceptible to serum killing (**Figure 3B**). Intracellular survival experiments in macrophages showed that SCV had the strongest ability to survive within macrophages, while the mucoid type showed a decreased ability to survive within macrophages (**Figure 3C**). In addition, we also conducted a biofilm formation experiment and found that the biofilm formation ability of SCV was the poorest, but the ability of biofilm formation was improved when SCV was transformed into mucoid type colonies (**Figure 3D**).

### Whole-genome sequencing analysis

**Figure 4:**
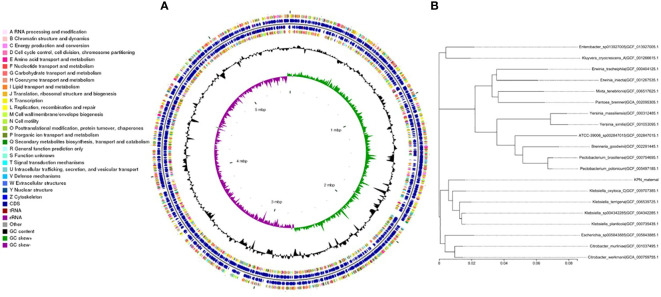
**(A)** Whole-genome sequencing analysis of maternal strain. **(B)** Phylogenetic tree of house-keeping genes of maternal strain.

**Figure 5:**
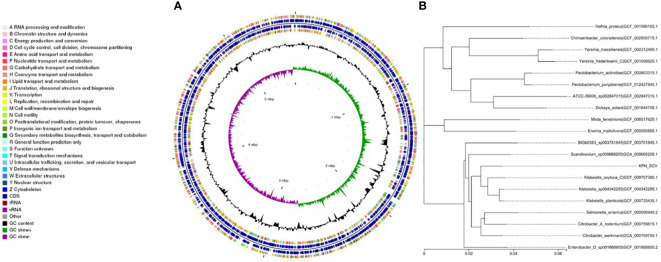
**(A)** Whole-genome sequencing analysis of SCV. **(B)** Phylogenetic tree of house-keeping genes of SCV.

**Figure 6:**
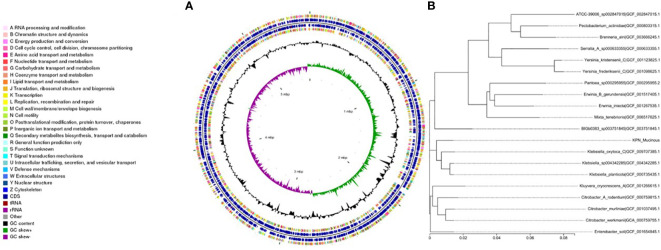
**(A)** Whole-genome sequencing analysis of mucoid type. **(B)** Phylogenetic tree of house-keeping genes of mucoid type.

We performed whole-genome sequencing analysis of the parental strain, SCV, and mucoid strain using third-generation sequencing technology. Based on the housekeeping gene sequences, a phylogenetic tree was constructed for the three strains, which showed that the closest strain to the three strains was Klebsiella_oxytoca_C/GCF_009707385.1 with a similarity of 99%. The circular genome map of the parental strain is shown in **Figure 4**. The genome size of the parental strain is 5541695 bp, with an average GC content of 56.98%. The genome size of SCV is 5542452 bp, with an average GC content of 57.01% (**Figure 5**). The genome size of mucoid strain is 5538988 bp, with an average GC content of 56.99% (**Figure 6**). All three strains have two plasmids, IncFIB(K) and IncFII(K).

### Analysis of virulence genes in the maternal strain, SCV, and mucoid strain

**Figure 7:**
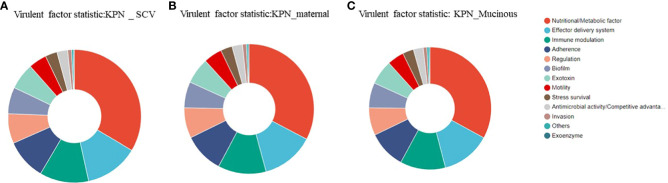
**(A)** Analysis of virulence genes in the maternal strain. **(B)** Analysis of virulence genes in SCV. **(C)** Analysis of virulence genes in the mucoid strain.

We analyzed the virulence system of the three strains (**Figure 7**) and found that SCV had a significantly higher number of nutritional/metabolic factors than the maternal strain and mucoid strain. While, the mucoid strain had an additional Exotoxin gene compared to SCV and the maternal strain (**Table 2**). At the same time, transporter protein analysis showed that the number of transporter protein genes in mucoid strain was much higher than that in SCV and the maternal strain (**Supplementary Table 1**). In addition, methylation analysis showed that both the maternal strain and mucoid strain had methylation modifications of 6mA and 4mC, while SCV lacked the 4mC methylation modification type (**Supplementary Table 2**). Further differential gene analysis of three strains revealed that the mucoid strain contained DNA polymerase V subunit UmuC gene on the plasmid (**Supplementary Table 3**), while the SCV strain had an additional IcmK family IV secretion protein on its plasmid (**Supplementary Table 4**).

**Table 2:** Analysis of virulence genes in the maternal strain, SCV, and mucoid strain.

Strains	maternal strain	SCV	mucoid strain
Nutritional/Metabolic factor	237	245	239
Effector delivery system	95	95	94
Immune modulation	87	87	87
Adherence	72	72	72
Regulation	55	53	53
Exotoxin	47	47	48
Biofilm	47	47	47
Motility	33	32	32
Stress survival	21	21	21
Antimicrobial activity	19	19	19
Invasion	7	7	7
Others	3	3	4
Exoenzyme	2	2	2

## Discussion

The incidence and severity of childhood pneumonia or lower respiratory tract infection (LRTI) have declined substantially in the last decade, but LRTI remains a major cause of mortality in children under five years of age, particularly in low- and middle-income countries (LMICs)(Collaborators, G.B.D.L.R.I, 2018). A retrospective cross-sectional study of 410 South African children hospitalized with KP-bacteremia found that 27% died within 30 days of infection (Buys et al., 2016). Conjugate vaccines (pneumococcal conjugate [PCV], and *Haemophilus influenzae type* b [Hib] vaccines), have reduced the burden of pneumonia and changed the etiological spectrum (Ning et al., 2017; Pneumonia Etiology Research for Child Health Study, G, 2019; Zar et al., 2022). KPN has been reported to be an important bacterial pathogen that causes neonatal sepsis and mortality, predominantly in southeast Asia (Kurade et al., 2018; Negash et al., 2019; Zar et al., 2022). Even more worrying, recurrent LRTI caused by Klebsiella pneumoniae was also on the rise (Karmaus et al., 2008). Recurrent or persistent infections can expose patients to antibiotics for a long time, this in turn leads to the development of subpopulations of antibiotic persisters (persister cells) that are not susceptible to antibiotics (Kaldalu et al., 2020).

The ability of persisters to survive their interaction with a host is important yet under investigated. Stapels et al. found that *Salmonella* persisters reprogram macrophages by means of effectors secreted by the *Salmonella* pathogenicity island 2 type 3 secretion system (Stapels et al., 2018). Another study had reported that Purine metabolism regulates *Vibrio splendidus* persistence associated with protein aggresome formation and intracellular tetracycline efflux (Li et al., 2023). Ernst et al. demonstrate that the mutants of wzc gene would lead to drug-tolerant K. pneumoniae and could persist to yield potentially untreatable, persistent infection (Ernst et al., 2020). In this study, we isolated SCV from children with recurrent LRTI and speculated that SCV was the reason for the child’s recurring pneumonia.SCV bacteria are commonly found in clinical infections, especially chronic and difficult-to-treat infections such as osteomyelitis, chronic respiratory infections, and cystic fibrosis (Kahl et al., 2016). SCV is a variant form of bacteria that forms smaller colonies on nutrient-rich media. Compared with normal strains, SCV grows slowly and is difficult to detect. Therefore, the epidemiology of SCV bacteria is of great significance. Currently, the epidemiology of SCV bacteria has not been fully studied. It had reported that long-term use of antibiotics may lead to the formation and transmission of SCV bacteria. Antibiotics can kill normal strains, but for SCV bacteria, because of their slow growth, antibiotics may not be able to completely eliminate them, leading to their proliferation in the patient’s body (Proctor et al., 2014; Zelmer et al., 2022). In addition, SCV bacteria may have the ability to adapt to adverse environments, making them easy to transmit in some specific environments(Proctor et al., 2014). Therefore, we speculate that recurrent lower respiratory tract infections in children are associated with the presence of SCV.

At present, the mechanism of SCV formation is still under study. Pitton et al. had confirmed the causative role of a single *ispA* mutation in SCV emergence and increased aminoglycoside resistance. IspA is involved in the synthesis of ubiquinone, providing a possible link between electron transport and SCV formation in P. aeruginosa (Pitton et al., 2022). Kleinert et al. inserted the IS256 sequence into the wild strain and found a very rapid reversion to the wild type that resembled the fast reversion of clinical SCVs (Kleinert et al., 2017). Islam et al. thought that iron restriction induces the small-colony variant phenotype in Staphylococcus aureus (Islam et al., 2022).

In this experiment, we found that the SCV of KPN had weaker resistance to serum, but was more likely to survive within macrophages. Differential gene analysis showed that the plasmid of the SCV had an additional IcmK family type IV secretion protein compared to the parental strain. The IcmK family type IV secretion proteins are a group of proteins that play important roles both inside and outside of cells. In bacteria, these proteins can be used to inject effector proteins, toxins, and other molecules into host cells, thereby influencing the physiological processes of the host cells(Daveri et al., 2023). Studies have shown that the IcmK family type IV secretion proteins play important roles in the pathogenicity, intracellular survival, and immune evasion of bacteria (Gomez et al., 2013). For example, a study on Pseudomonas aeruginosa found that IcmK family type IV secretion proteins could enhance the survival of this bacteria within host cells, and could also inhibit the apoptotic response of host cells, thereby increasing its virulence (Persat et al., 2015). In addition, Coxiella burnetiid, which causes Q fever, also uses a Dot/Icm-like system to secrete effector proteins via IcmK family type IV secretion proteins. These effectors can modulate host cell processes such as apoptosis, autophagy, and inflammation, allowing the bacterium to establish a replicative niche within the host cell (Segal et al., 2005). Therefore, we speculate that IcmK family type IV secretion proteins may help the SCV of KPN to survive within host cells, thereby enhancing its intracellular survival ability.

In addition, we also found that the SCV of KPN changed to a mucoid colony when exposed to subinhibitory concentrations of ciprofloxacin. A study had reported that Salmonella enterica persister cells form unstable small colony variants after *in vitro* exposure to ciprofloxacin (Drescher et al., 2019). However, this article does not explain the mechanism. To explain the underlying mechanism of this phenomenon, we conducted whole-genome sequencing analyses of SCV and mucoid colonies.Differential gene analysis revealed that the plasmid of the mucoid-type colony carried DNA polymerase V subunit UmuC gene. UmuC is an important component of the DNA damage-induced mutagenic repair mechanism, and its main function is to participate in the DNA damage repair process (Kuban et al., 2012). When exposed to a DNA damage source, the expression level of UmuC in Klebsiella pneumoniae is upregulated to cope with the DNA damage. As DNA damage repair requires the consumption of cellular energy and resources, and may interfere with normal DNA replication and cell division processes. This may be the reason why the mucoid-type Klebsiella pneumoniae grows slower than the parental strain. In addition, some studies have found that UmuC is quite related to the SOS response, which involved in immune evasion and serum resistance in bacteria (Sutton et al., 2000). Specifically, UmuC may regulate bacterial recognition and evasion of host serum by affecting the surface structure, cell wall composition, and intracellular protein expression of bacteria (Timinskas and Venclovas, 2019; Tanooka et al., 2020). Some studies had demonstrated that *umuC* gene involved in the formation of tigecycline resistance mechanisms as the potential insertion site of *tmexCD1-toprJ1* (Peng et al., 2021; Sun et al., 2022). Although further research is needed to fully understand the specific mechanisms and effects of UmuC on bacterial serum resistance, existing studies suggest that UmuC may be an important regulatory factor in bacterial immune evasion and antibiotic resistance.

## Conclusion

Our study showed the SCV of KPN changed to a mucoid colony when exposed to subinhibitory concentrations of ciprofloxacin. Mucoid colonies had a higher resistance of serum, which was possibly related to the UmuC gene. The increased intracellular survival of SCV may be related to the IcmK family type IV secretion proteins.

## Data availability statement

The data presented in the study are deposited in the NCBI repository, accession number: SAMN36887619, SAMN36887620, SAMN36887621.

## Ethics statement

The data and samples analyzed in the present study were obtained in accordance with the standards and approved by Chongqing Health Center for Women and Children the Institutional Review Board and Biomedical Ethics Committee. For this study, samples were collected at the microbiology laboratory of our hospital, with no contact with the patient. This study was retrospective and there was no patient identification performed during data collection. Therefore, the ethics committee determined that informed consent was not required.

## Author contributions

HZ: Writing – original draft, Writing – review & editing. QL: Methodology, Writing – original draft. JH: Methodology, Writing – original draft. CL: Writing – original draft. YS: Conceptualization, Writing – original draft. LZ: Formal Analysis, Writing – original draft. XZ: Data curation, Writing – original draft.
